# Genomic Loss of Tumor Suppressor miRNA-204 Promotes Cancer Cell Migration and Invasion by Activating AKT/mTOR/Rac1 Signaling and Actin Reorganization

**DOI:** 10.1371/journal.pone.0052397

**Published:** 2012-12-21

**Authors:** J. Saadi Imam, Jason R. Plyler, Hima Bansal, Suresh Prajapati, Sanjay Bansal, Jennifer Rebeles, Hung-I Harry Chen, Yao-Fu Chang, Subbarayalu Panneerdoss, Behyar Zoghi, Kalyan C. Buddavarapu, Russell Broaddus, Peter Hornsby, Gail Tomlinson, Jeffrey Dome, Ratna K. Vadlamudi, Alexander Pertsemlidis, Yidong Chen, Manjeet K. Rao

**Affiliations:** 1 Greehey Children’s Cancer Research Institute, University of Texas Health Science Center at San Antonio, San Antonio, Texas, United States of America; 2 Department of Cellular and Structural Biology, University of Texas Health Science Center at San Antonio, San Antonio, Texas, United States of America; 3 Department of Medicine, University of Texas Health Science Center at San Antonio, San Antonio, Texas, United States of America; 4 Department of Physiology, University of Texas Health Science Center at San Antonio, San Antonio, Texas, United States of America; 5 Department of Obstetrics and Gynecology, University of Texas Health Science Center at San Antonio, San Antonio, Texas, United States of America; 6 Department of Epidemiology and Biostatistics, University of Texas Health Science Center at San Antonio, San Antonio, Texas, United States of America; 7 Department of Pathology, University of Texas MD Anderson Cancer Center, Houston, Texas, United States of America; 8 Center for Cancer and Immunology Research, Children’s National Medical Center, Washington D.C., United States of America; University Magna Graecia, Italy

## Abstract

Increasing evidence suggests that chromosomal regions containing microRNAs are functionally important in cancers. Here, we show that genomic loci encoding miR-204 are frequently lost in multiple cancers, including ovarian cancers, pediatric renal tumors, and breast cancers. MiR-204 shows drastically reduced expression in several cancers and acts as a potent tumor suppressor, inhibiting tumor metastasis in vivo when systemically delivered. We demonstrated that miR-204 exerts its function by targeting genes involved in tumorigenesis including *brain-derived neurotrophic factor* (*BDNF*), a neurotrophin family member which is known to promote tumor angiogenesis and invasiveness. Analysis of primary tumors shows that increased expression of BDNF or its receptor tropomyosin-related kinase B (TrkB) parallel a markedly reduced expression of miR-204. Our results reveal that loss of miR-204 results in BDNF overexpression and subsequent activation of the small GTPase Rac1 and actin reorganization through the AKT/mTOR signaling pathway leading to cancer cell migration and invasion. These results suggest that microdeletion of genomic loci containing miR-204 is directly linked with the deregulation of key oncogenic pathways that provide crucial stimulus for tumor growth and metastasis. Our findings provide a strong rationale for manipulating miR-204 levels therapeutically to suppress tumor metastasis.

## Introduction

Identification of chromosomal regions harboring oncogenes and tumor suppressor is crucial for understanding of the tumor pathogenesis and improved treatment outcomes [1]. Although several cancer-related genes have been identified in regions with chromosomal abnormalities [2], additional regions with random or recurrent chromosomal abnormalities harboring factors important in cancer growth and progression remain to be identified. Recent studies indicate that microRNAs (miRNAs) are one such group of factors [3]. MiRNAs are small, endogenous, non-coding RNAs that regulate post-transcriptional gene expression by binding to 3′ untranslated regions (UTRs) of target mRNAs. MiRNAs are known to play crucial functions in multiple biological processes including development and differentiation [4], and deregulated expression of miRNAs has been implicated in several human diseases including cancer [5]. Growing evidence suggests that miRNAs can act as oncogenes or tumor suppressor genes [6].

In this study, we focused on chromosomal loci containing miR-204. MiR-204 has been reported to play important roles in smooth muscle cell calcification as well as endoplasmic reticulum stress response in trabecular meshwork cells [7], [8]. In addition, recent reports have shown equally important roles for miR-204 in tumorigenesis, including regulation of carcinogenesis in peripheral nerve sheath tumors, and migration and invasion of endometrial cancer cell lines [9], [10]. However, very little is known about the mechanism by which miR-204 regulates oncogenesis in general, and tumor growth and metastasis in breast and ovarian cancers in particular. Furthermore, virtually nothing is known about whether miR-204 can be targeted therapeutically and how miR-204 expression is regulated in cancers. Our results reveal that chromosomal loci containing miR-204 is frequently lost, resulting in its lower expression in multiple cancers. We demonstrate that miR-204 acts as a potent tumor growth and metastasis suppressor. Importantly, our results show that miR-204 can be targeted therapeutically as systemic delivery of miR-204 inhibited breast cancer lung metastasis without causing any hepatotoxicity. We found that miR-204 acts as a tumor suppressor by targeting the function of genes associated with tumorigenesis, including *brain-derived neurotrophic factor* (*BDNF*). BDNF is a physiologically important nerve growth factor that plays a critical role in the development of nervous system by binding and subsequently activating the tyrosine kinase receptor, tropomyosin-related kinase B (TrkB) [11], [12]. In addition, the BDNF/TrkB pathway is reported to have a critical role in tumorigenesis as it promotes proliferation, differentiation, angiogenesis and tumor invasiveness [13]. Overexpression of BDNF/TrkB has also been implicated in poor prognosis of several solid tumors including neuroblastoma, ovarian, breast, prostate and lung cancers [14], [15], [16]. Our findings reveal that loss of miR-204 results in BDNF/TrkB overexpression and concomitant activation of the AKT/mTOR/Rac1 signaling pathway, leading to actin reorganization during cancer cell migration and invasion. These results underline the functional importance of chromosomal regions containing specific miRNAs in tumor growth and metastasis.

## Results

### Somatic Loss of miR-204 in Cancers

**Figure 1:**
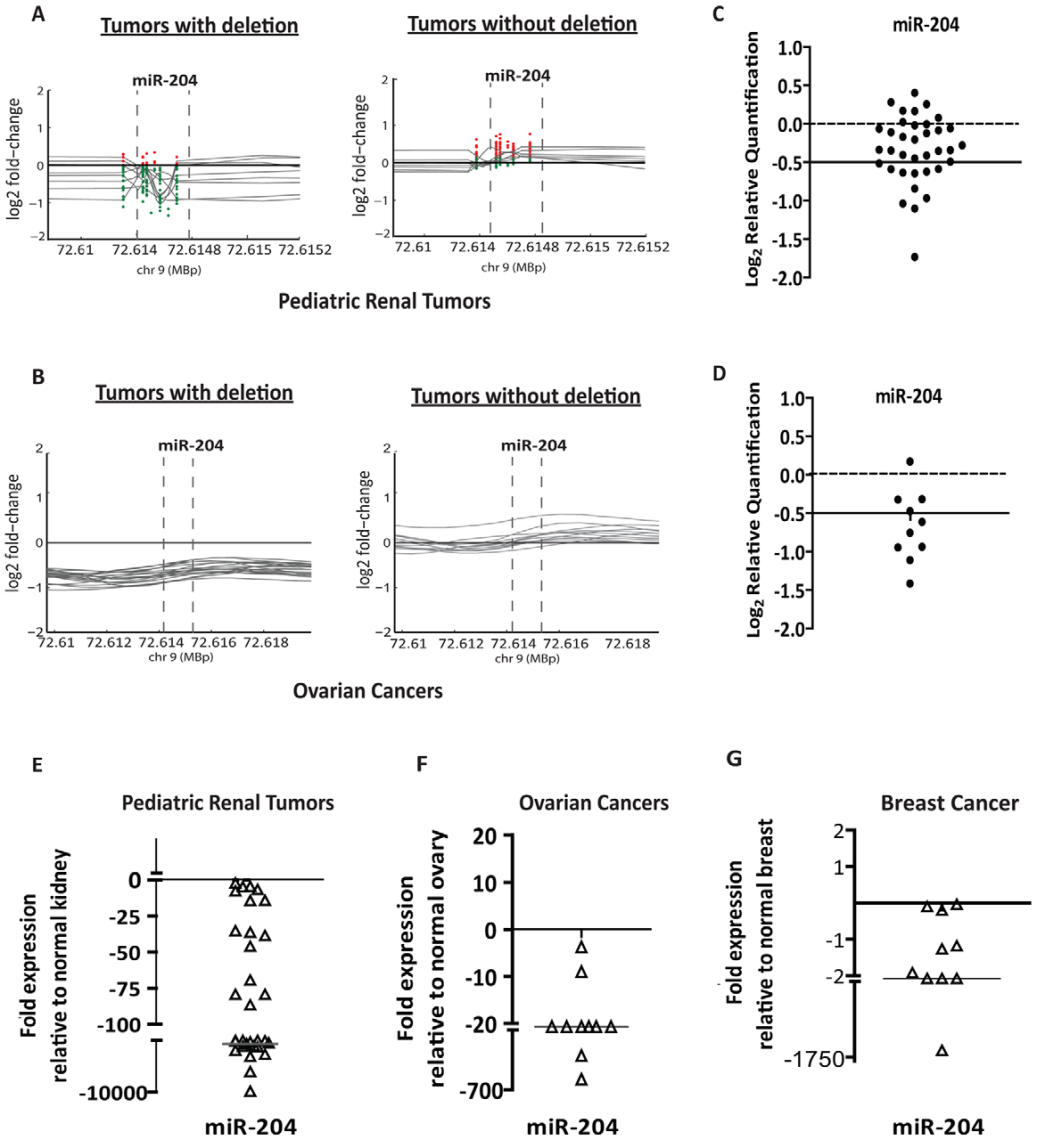
Genomic loss of miR-204 in cancers. A, high resolution miRNA-CGH on selected pediatric renal tumors with (left) or without (right) miR-204 deletions. Deletion of genomic loci containing the miRNA is indicated by dotted perpendicular lines. Red and green dots indicate position and value of each probe reflecting copy number change, represented in triplicate on the CGH array. The grey trend line represents the average value of the triplicate probe for each tumor. B, graphs obtained from meta-analysis of high-resolution CGH of ovarian cancers (*n* = 354; obtained from TCGA) representing a subset of tumors with or without deletion. The deletion of genomic loci containing miR-204 is indicated by dotted perpendicular lines. C and D, allelic PCR of miR-204 genomic locus in pediatric renal tumors (C) and ovarian cancers (D). The y-axis shows log_2_ transformed relative quantification values. Dotted lines show the loss of copy threshold. E–G, graphical representation of qRT-PCR analysis showing levels of miR-204 in pediatric renal tumors (*n* = 38; E), in advanced stage ovarian cancers (*n* = 11; F) and, in breast cancers (*n* = 10; G), when compared to normal matched control kidney (*n* = 38), normal ovarian tissues (*n* = 5) and normal matched breast tissues (*n* = 10).

To identify miRNAs that are associated with aberrant chromosomal regions in human cancers, we used high-resolution custom miRNA comparative genomic hybridization (CGH) and high-density CGH public domain datasets for ovarian cancers, breast cancers and pediatric renal tumors. This analysis revealed several miRNAs in the minimal chromosomal deletion and amplification regions of these tumors. To begin to understand the functional importance of chromosomal loci associated with miRNAs in tumorigenesis, we initially focused on miRNAs exhibiting genomic loss (Figure S1A). The 9q21.12 chromosomal region containing miR-204 was frequently lost in 44.63% (158/354) of ovarian cancers, 28% (10/35) of breast cancers and 40% (15/38) of pediatric renal tumors (Figure 1A, 1B, and Figure S1B), which is further verified by quantitative genomic real-time PCR analysis (Figure 1C and 1D). Furthermore, reduction in the levels of mature miR-204 also strongly correlated with its genomic DNA content in all three tumor types (Figure 1E–1G).

### MiR-204 Acts as a Potent Tumor Growth and Metastasis Suppressor

**Figure 2:**
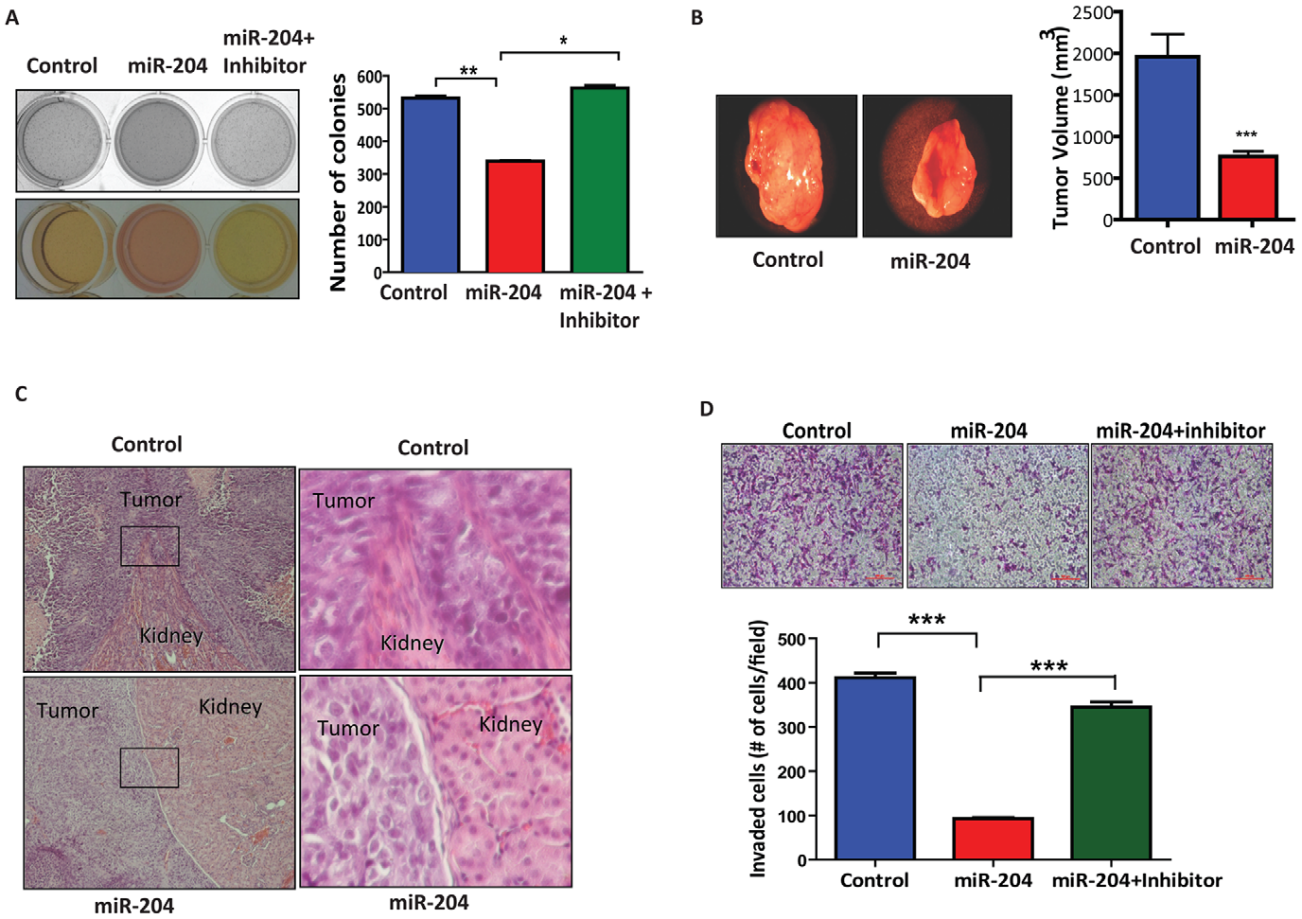
MiR-204 inhibits tumor growth and metastasis. A, miR-204 inhibits anchorage-independent growth. Nutrient consumption (left) and graph (right) depict the number of colonies formed in soft agar wells by HEK-293 cells stably overexpressing either scramble or miR-204, further transfected with miR-204 inhibitor. (*) *P*<0.05; (**) *P*<0.01. Results are the average of three independent experiments. B, miR-204 overexpression inhibits tumor growth. Photograph shows representative features of tumor growth in *RAG2* ^−/−^, γc^−/−^ SCID mice injected (in the kidney capsule) with HEK-293 cells stably overexpressing either scramble control or miR-204. Bar graph shows mean tumor volume for miR-204 (*n* = 9) and scramble (*n* = 9) transfectants. (*) *P*<0.001. C, histological analysis of sections from tumor xenografts overexpressing either scramble (control) or miR-204. Images shown in the right panel represent magnified view of boxed region indicated in the left panel. Tumor invasion in control transfectants is reflected by the invasion of tumor into renal tissue. D, basement membrane matrix invasion assay of MDA-MB-231 cells transfected with 75 nM scrambled sequence (control) or miR-204 mimic (miR-204) or miR-204 mimic transfected cells further transfected with miR-204 inhibitor (miR-204+inhibitor). Bar graph shows the average number of invaded cells counted microscopically in five different fields per filter. (***) *P*<0.001.

The drastically reduced expression of miR-204 in multiple cancer tissues prompted us to address the role of miR-204 in tumorigenesis. To determine this, we first assessed the effect of miR-204 on anchorage independent growth. Embryonal kidney HEK-293 cells (HEK-293 cells passaged over 52 times are reported to be highly tumorigenic [17]) overexpressing miR-204 exhibited reduced colony-forming capacity, which was rescued with the overexpression of an inhibitor of miR-204 (Figure 2A). Similar results were also obtained with miR-204 overexpressing breast cancer MDA-MB-231 and ovarian cancer SKOV3 cells (data not shown). To further confirm miR-204’s potential tumor suppressor-like activity, high-passage HEK-293 cells stably overexpressing either miR-204 or a scrambled sequence were injected into the kidney capsules of nude mice and tumor growth and metastasis were evaluated 24 days after injection. In sharp contrast to control tumors, miR-204 overexpressing tumors were dramatically reduced in size (Figure 2B). Next, we examined whether miR-204 also inhibits tumor cell invasion. Histological analysis of xenograft tumor sections indicated drastically decreased or no invasiveness of tumors into renal tissues that overexpressed miR-204 compared to control (Figure 2C). Consistent with this, miR-204 overexpression drastically reduced the migratory and invasive capabilities of breast cancer (MDA-MB-231) and ovarian cancer (SKOV3) cells (Figure 2D and data not shown) in vitro.

### Therapeutic Targeting of miR-204

**Figure 3:**
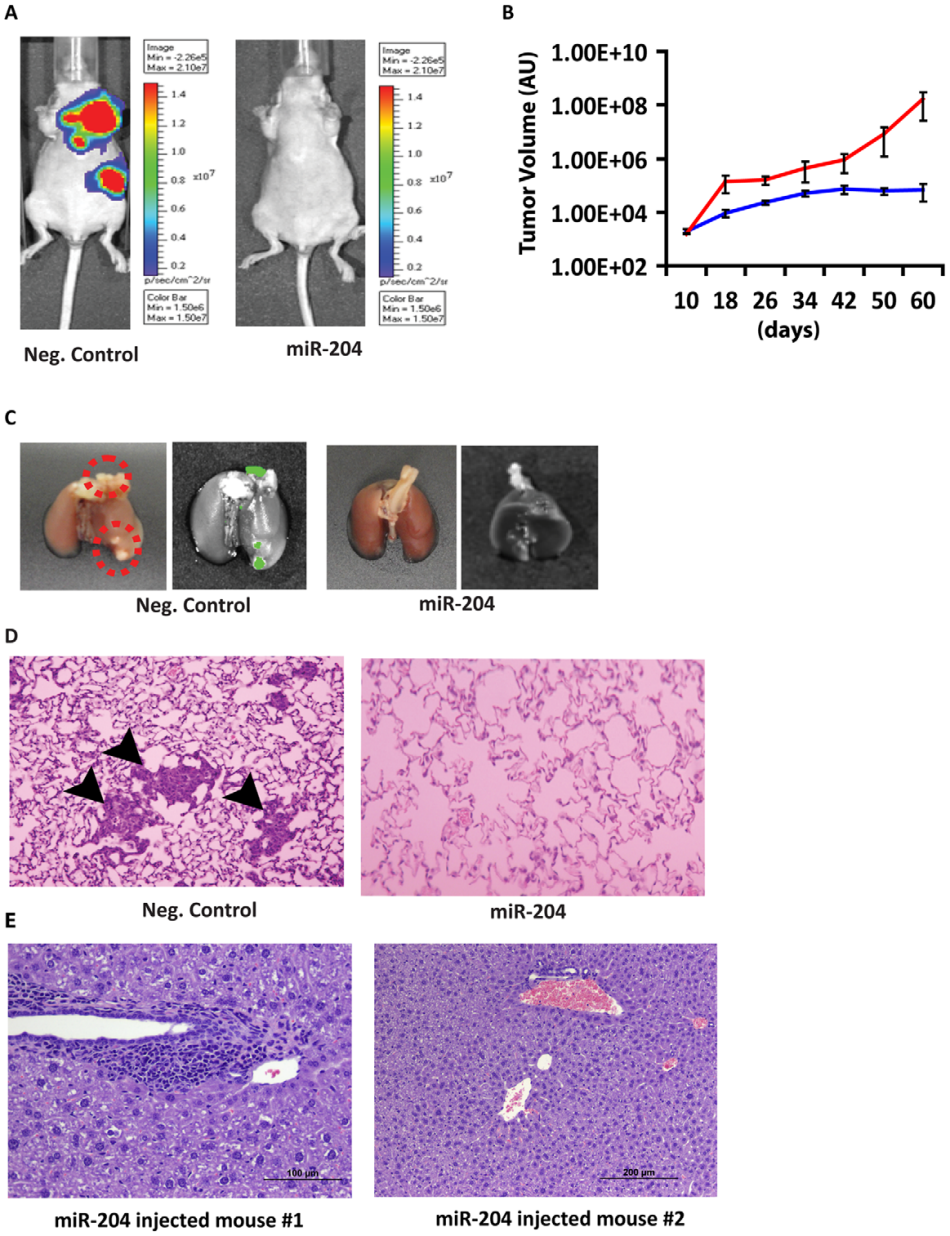
Systemic delivery of miR-204 suppresses tumor metastasis. A, injection of miR-204 oligonucleotide into tail vein suppressed lung metastasis. Live bioluminescence images of mice injected with miR-204 (*n* = 6) or miR-204 mutant (neg. control; *n* = 6) oligonucleotides using the Xenogen In Vivo Imaging System (IVIS) (Xenogen). Images were taken after subcutaneously injecting 150 mg/kg D-luciferin substrate in PBS to anesthetized mice. B, tumor metastasis volume was assessed starting from day 10 until animals were sacrificed at day 60. Using ROI analysis, tumor light intensity was calculated in photon/s, which corresponds with the number of live cells in vivo. C, representative lung images showing GFP^+ve^ foci (red circle) in neg. control groups. D, representative lung sections showing metastatic foci in neg. control groups. E, no hepatotoxicity in miR-204 injected mice. Sections of liver from miR-204 injected mice show no signs of hepatotoxicity. The presence of multifocal periportal lymphocytes is not unusual and is a common finding in young animals.

To further substantiate the metastasis suppressor activity of miR-204 we performed therapeutic experiments in the breast cancer lung metastasis model. We first established lung metastases by tail vein injection of MDA-MB-231 breast cancer cells expressing luciferase-GFP, and subsequently injected miR-204 or miR-204 mutant oligo (negative control) into the tail vein of nude mice every 5 days for 30 days using LANCErII, a lipid-based in vivo delivery vehicle. Interestingly, systemic delivery of miR-204 resulted in significant reduction or elimination of lung metastases, while miR-204 mutant oligo injected mice had severe lung metastasis, as measured over the 60-day period (Figure 3A–3D and Figure S2). Importantly, mice injected with miR-204 showed no obvious side effects as revealed by absence of hepatotoxicity or changes in body weight (Figure 3E and data not shown). These results indicate that miR-204 can be a safe and viable therapeutic regimen to treat tumor growth and metastasis.

### MiR-204 Targets Genes Associated with Tumorigenesis

**Figure 4:**
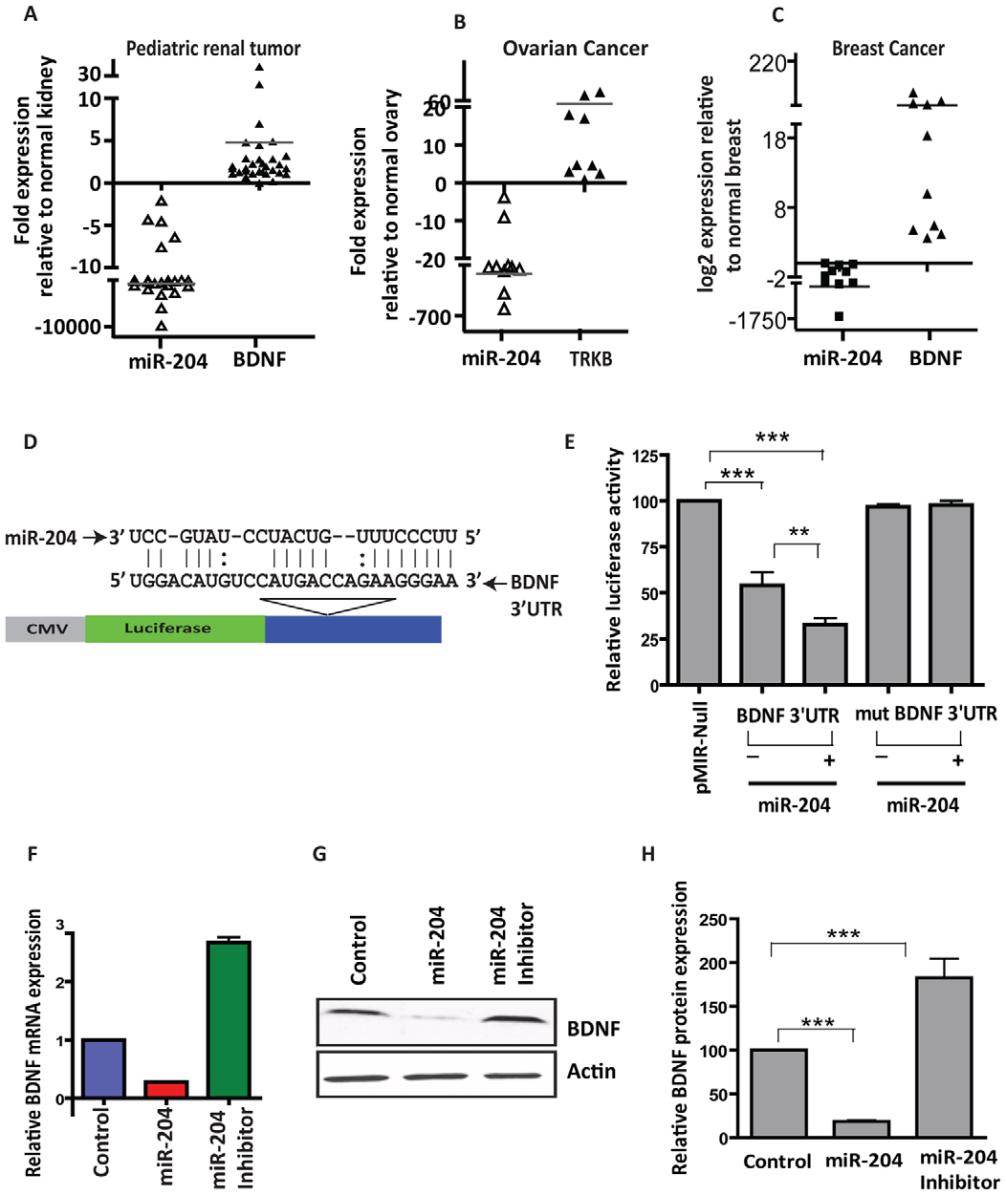
MiR-204 regulates expression of BDNF in cancers. A–C, increased BDNF expression correlates strongly with lower miR-204 expression in multiple cancers. Graphical representation of qRT-PCR analysis showing the inverse correlation between miR-204 and *BDNF* in pediatric renal tumors (*n* = 38; A), advanced stage ovarian cancers (*n* = 11; B) and breast cancers (*n* = 10; C), compared to normal matched control kidney (*n* = 38), normal ovarian tissues (*n* = 5) and normal matched breast tissues (*n* = 10). D–H, *BDNF* is a bonafide target of miR-204. D, schematic of the putative miR-204 binding sequence in the *BDNF* 3′ UTR. E, HEK-293 cells were co-transfected with Renilla luciferase expression construct pRL-TK and firefly luciferase constructs containing either pMIR-*BDNF* 3′ UTR in the absence and presence of miR-204 mimic or pMIR-*BDNF* 3′ UTR mutant. Firefly luciferase activity of each sample was normalized to Renilla luciferase activity. Mean±SEM of three independent experiments (performed in duplicate for each experiment). (**) *P*<0.01; (***) *P*<0.001. F, qRT-PCR analysis of miR-204 overexpressing cells and cells transfected with miR-204 inhibitors using *BDNF*-specific primers. G, western blot analysis of HEK-293 cells transfected with miR-204 mimic using anti-BDNF antibody (1∶1000). β-actin was used as a loading control. Gel photographs are representative of three independent experiments. H, graphical representation of band intensities quantified using the Total Labs TL100 1D gel analysis software (*n* = 3; Nonlinear). BDNF protein level for the control was set to 100.

To understand the mechanism by which miR-204 may play a role in tumorigenesis, we identified genes regulated by miR-204. Since most miRNAs act to decrease target mRNA levels [18], we performed gene expression analyses on cells overexpressing miR-204 and determined the potential targets of miR-204. Of the genes altered in miR-204 overexpressing cells, downregulated genes are most likely to be directly targeted by miR-204. Interestingly, several of the downregulated genes are associated with cancer-related processes and physically or functionally interact with each other, as revealed by pathway-based analyses (Figure S3). We performed detailed analyses on two such genes: *BDNF* and *Ezrin* that showed higher levels of alteration in our microarray analysis and featured in all predicted biological pathways with highest functional enrichment significance. BDNF with its receptor TrkB is known play a critical role in tumor angiogenesis and metastasis; and Ezrin, a cytoskeletal organizer protein, has been implicated in the tumor growth metastasis of several adult and pediatric tumors including osteosarcoma, mammary and pancreatic adenocarcinomas, ovarian carcinoma as well as rhabdomyosarcoma and pediatric renal tumors [19], [20], [21]. Moreover, multiple target prediction algorithms, including SvMicrO [22], Bayesian decision fusion approach [23], and miRmate [24] that we have recently generated as well as TargetScan [25] and Pictar [26], also predicted *BDNF* and *Ezrin* to be targeted by miR-204. Furthermore, miR-204 binding sites were found to be evolutionarily conserved throughout vertebrates, suggesting it to have an important regulatory function across a variety of species. Importantly, miR-204 and *BDNF*/*TrkB* as well *Ezrin* expression showed a strong inverse correlation in several tumors (Figure 4A–4C and Figure S4A–S4C).

To validate our microarray and target prediction results, we first examined whether miR-204 targets *BDNF*/*Ezrin* by binding to the predicted site in its 3′ UTR (Figure 4D and Figure S4D). Indeed, the luciferase activity of a pMIR-reporter construct containing the *BDNF* or *Ezrin* 3′ UTR was significantly repressed, which was further reduced in cells overexpressing miR-204 when compared to construct without the 3′ UTR (Figure 4E and Figure S4D). In contrast, mutation of the seed sequence in *BDNF* 3′ UTR-containing construct not only restored luciferase activity to near that of the wild-type construct but also rendered transcripts from these constructs insensitive to miR-204 overexpression (Figure 4E), confirming a specific interaction between miR-204 and the predicted binding site in the 3′ UTRs of these genes. To further substantiate these results, we determined the levels of BDNF/Ezrin in cells overexpressing miR-204. MiR-204 overexpression resulted in significant reduction of BDNF/Ezrin both at the RNA and protein levels (Figure 4F–4H and Figure S4E and S4F). Next, we examined whether or not BDNF and Ezrin are functionally important targets of miR-204. To address that we performed rescue experiments. Reintroduction of *BDNF* or *Ezrin* rescued miR-204 induced phenotypes including anchorage-independent growth, cell migration and invasion (Figure S5 and data not shown). These results suggest that miR-204-mediated regulation of *BDNF* and *Ezrin* is an important event in cancer cell growth, migration and invasion.

### Loss of miR-204 Activates AKT/mTOR Signaling and Rac1 Translocation in Cancer Cells

**Figure 5:**
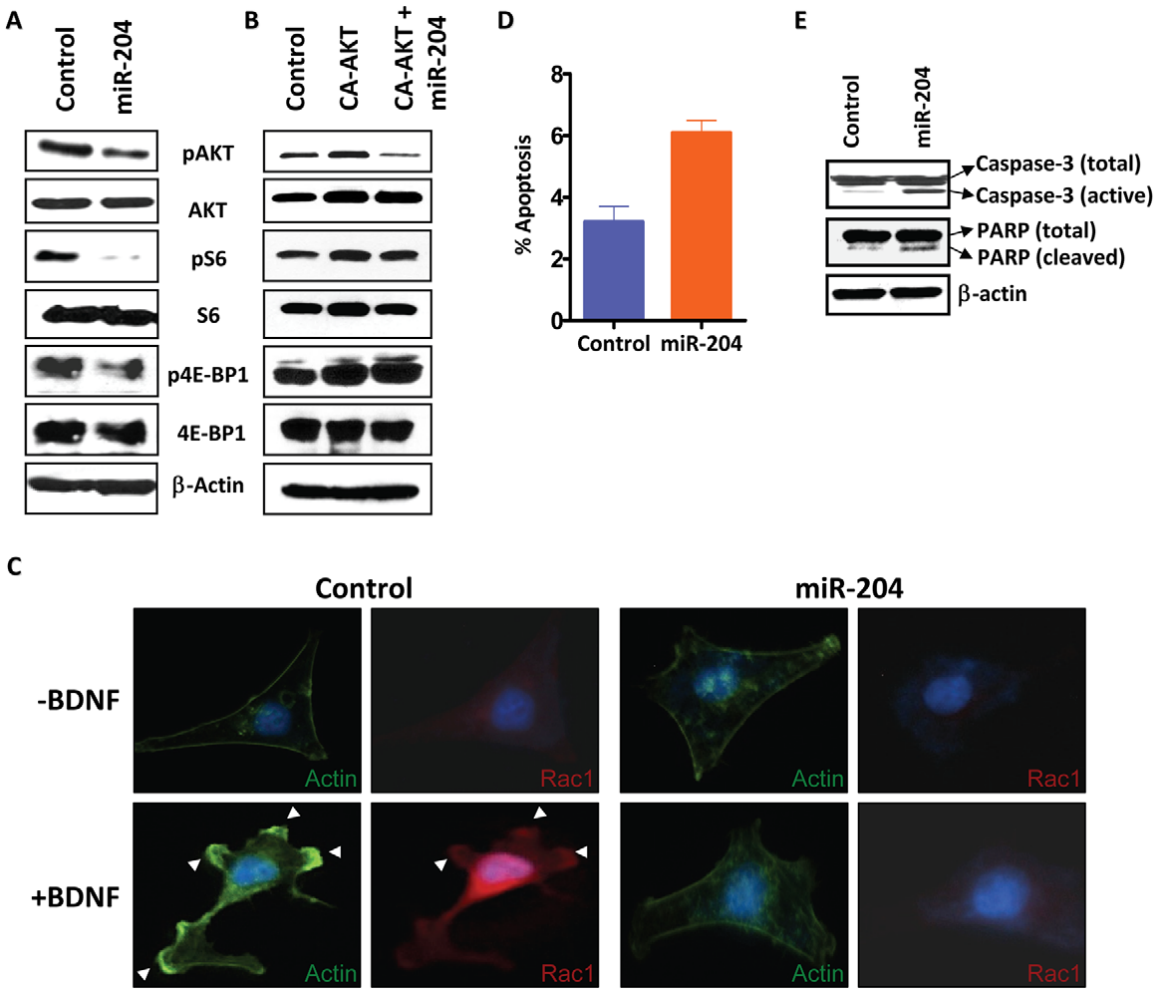
MiR-204 inhibits tumor cell migration and invasion by altering AKT/mTOR/Rac1 signaling. A, miR-204 suppresses activation of AKT and mTOR signaling. HEK-293 cells transfected with miR-204 were grown in serum-free conditions and subjected to western blot analysis using anti-phospho-Ser^473^-AKT (1∶1000), anti-total-AKT (1∶1000), anti-phospho-Ser^235/236^-S6 (1∶1000), anti-total-S6 (1∶1000), anti-phospho-Thr^37/46^-4E-BP1 (1∶1000) and anti-total-4E-BP1 (1∶1000). β-actin (1∶10,000) was used as a loading control. Gel photographs are representative of three independent experiments. B, HEK-293 cells transfected with constitutively active AKT T308D/S473D mutant (CA-AKT) in the absence and presence of miR-204 were grown in serum free condition and subjected to western blot analysis as descried in A. C, overexpression of miR-204 abolishes BDNF-induced membrane ruffling and Rac1 translocation. HEK-293 cells transfected with miR-204 or miR-185 were grown in serum-free conditions and treated with or without BDNF (100 ng/mL) for 10 min and stained with Rac1 antibody (red) and FITC-phalloidin (green). Arrows indicate membrane-ruffling regions. D, miR-204 increases the sensitivity of HEK-293 cells to apoptosis as determined by Annexin V/PI staining using the FITC-Annexin V Apoptosis Detection Kit. The percentage cell population shown is the mean±SEM of three independent experiments. E, western blot analysis of HEK-293 cells transfected with miR-204 using anti-caspase-3 (1∶500) antibody and anti-PARP (1∶1000) show increased cleavage of caspase-3 and PARP. β-actin (1∶10,000) was used as a loading control. Gel photograph is representative of three independent experiments.

To determine the mechanism by which miR-204 may exhibit its tumor growth and metastasis suppressor activity, we examined the effect of miR-204 on AKT/mTOR signaling as both BDNF and Ezrin have been shown to activate AKT pathway [27], and selective activation of AKT by mTOR has been shown to regulate cancer cell migration and invasion [28]. Interestingly, miR-204 overexpression resulted in reduced activity of AKT and mTOR downstream targets 4E-BP1 and S6 (Figure 5A). 4E-BP1 is a translation inhibitor that dissociates from eIF4E upon phosphorylation to allow protein translation, and S6 is a ribosomal protein whose phosphorylation facilitates assembly of the ribosome and consequent translation of mRNA. To confirm our in vitro findings, we analyzed expression of phospho-AKT (pAKT) and phospho-S6 (pS6) in tumor xenograft overexpressing miR-204. As shown in Figure S6, the levels of pAKT and pS6 were significantly reduced, while total AKT and total S6 levels remain unchanged in tumors overexpressing miR-204 when compared to control transfectant tumors, as revealed by immunohistochemical analysis. To further substantiate the notion that the AKT/mTOR pathway is indeed an important target of miR-204, we co-transfected miR-204 with the constitutively active AKT T308D/S473D mutant and showed that constitutively active AKT rescued the negative effects of miR-204 on downstream pS6 and phospho-4E-BP1 (p4E-BP1) levels (Figure 5, compare 5A and 5B). However, we could not detect the rescue of negative effects mediated by miR-204 on pAKT levels in cells co-transfected with constitutively active AKT (Figure 5B). This is due to the mutation of Ser^473^ to Asp^473^ in constitutively active AKT, and therefore the anti-phospho-Ser^473^-AKT antibody used for the western blot analysis only detected endogenous pAKT, which is reduced in level in the presence of miR-204 (Figure 5A and 5B).

AKT controls cell invasiveness by regulating multiple processes that are involved in actin organization, cell-to-cell adhesion, and cell motility [28], [29], [30]. To examine whether miR-204 may play a direct role in this process, we assessed the effect of miR-204 overexpression on the activation of the small GTPase protein Rac1, which functionally interacts with AKT/mTOR and is reported to play a critical role in cell migration and actin reorganization upon induction with BDNF [31] or epidermal growth factor (EGF) [28]. Stimulation of MDA-MB-231 cells (and SKOV3 or HEK-293 cells) with BDNF or EGF induced membrane ruffling and caused Rac1 to be translocated to the ruffling region (Figure 5C, Figure S7, and data not shown). In contrast, the membrane ruffling and Rac1 translocation were not observed in cells overexpressing miR-204 when induced with BDNF or EGF (Figure 5C, Figure S7, and data not shown). Evasion of apoptosis, which is critical for tumor growth and progression [32] could be another mechanism central to oncogenesis in cancers exhibiting loss of miR-204. Moreover, increased AKT/mTOR activity has also been associated with decreased apoptosis in cancers [33]. Indeed, miR-204 overexpression caused a significant increase in the levels of both activated caspase-3 and poly ADP ribose polymerase (PARP), indicators of irreversible damage to the integrity of the cell and genome, with a resultant increase in apoptotic activity (Figure 5D and 5E). Taken together, our findings suggest that loss of miR-204 promotes tumor cell growth, migration and invasion by activating its target genes that are known regulators of oncogenic signaling cascade.

## Discussion

Many miRNAs associated with cancers are known to be localized to genomic fragile sites [34]. However, surprisingly little is known about the functional importance of regions with chromosomal aberrations containing miRNAs. It is likely that initial genetic screens (with lower comparative genomic hybridization resolution) aimed at discovering chromosomal abnormalities overlooked changes in genomic regions containing miRNAs. Because miRNAs influence several genes in one or more pathways that regulate cell growth and apoptosis and contribute to tumor formation when deregulated, a closer scrutiny of smaller genomic regions encoding miRNAs will likely provide important insights into the mechanism of tumorigenesis. Since most miRNAs are proposed to be downregulated in cancers, we believe that microdeletion of genetic regions containing specific miRNAs is a more frequent event that plays an important role in the development and progression of human cancers and merits greater attention.

In this study we provide evidence that miR-204, which acts as a potent tumor growth and metastasis suppressor, is somatically lost in human cancers. We demonstrate that miR-204 regulates the expression and function of pro-angiogenic protein BDNF and its receptor TrkB in tumors. Our results show that loss of miR-204 promotes BDNF (or EGF)-induced cancer cell migration and invasion by activating AKT/mTOR pathway leading to Rac1 translocation and actin reorganization. Since AKT and Rac1 require each other for their activation [28], [35], our findings suggest that loss of miR-204 expression in human tumors may induce the positive feedback loop between BDNF/AKT1/mTOR and Rac1 during cancer cell migration and invasion. Supporting this, both mTOR complexes mTORC1 and mTORC2 have been reported to regulate motility and metastasis of colorectal cancer via the Rac1 signaling pathway [36], and Rac1 has been shown to simultaneously regulate mTORC1 and mTORC2 [37]. Furthermore, BDNF, which activates GTP-bound Rac1 levels [38], has been shown to be blocked by mTOR inhibitor rapamycin [39]. These studies, along with our results showing reduced levels of pAKT/pS6K in cell lines and in tumors overexpressing miR-204, strongly indicate that BDNF/AKT/mTOR/Rac1 signaling cascade is one of the major targets of miR-204. Future studies aimed at addressing the effect of miR-204 on mTORC1 and mTORC2 components such as Raptor/Sin1 will likely provide additional insights. Similar to BDNF, our results show that miR-204 inhibits EGF-induced activation of Rac1. This is significant as crosstalk between EGFR and BDNF receptor TrkB has been shown to induce cancer cell migration [40] and response to EGF is a key step during cancer cell invasion and is directly linked with metastasis [41]. These observations suggest that miR-204 may have a broader role in altering growth factor-induced cancer cell migration and invasion in general. In addition to cancer cell migration and invasion, activation of BDNF/TrkB signaling may also contribute to evasion of apoptosis in miR-204 depleted cells as BDNF/TrkB overexpression has been linked with stabilization and activation of AKT resulting in decreased apoptosis [42]. Consistent with this, our results show that miR-204 overexpression results in reduced phosphorylation and activation of mTOR downstream targets 4E-BP1 and S6 kinase.

Because miR-204 resides within the *transient receptor potential cation channel member 3* (*TRPM3*) gene, which is a member of transient receptor potential channels family of proteins known to be important for cellular calcium signaling and homeostasis, it will be tempting to speculate that the phenotypes associated with the genomic loss of chromosomal loci containing miR-204 may also be contributed by the host gene *TRPM3*. Our results showing suppression of tumor growth and metastasis following introduction of miR-204 alone, and no change in the levels of *TRPM3* gene in miR-204 mimic or inhibitor overexpressing cancer cells (Figure S8), clearly suggest that miR-204 associated tumor suppressor phenotype is not mediated through *TRPM3*. However, we cannot exclude the possibility that miR-204 and its host gene *TRPM3* act synergistically to regulate suppression of tumor growth as well as tumor cell migration and invasion. Since a definite role for *TRPM3* in tumorigenesis has not been reported, a detailed study examining *TRPM3*’s potential tumor suppressor role and its synergistic effect with miR-204 (if any) will be subject of future investigations.

Taken together, our findings suggest that genetic loci containing specific miRNA may play a causal role in cancer growth and metastasis by regulating key oncogenic pathway. The ability of miR-204 to target *BDNF*/*TrkB* and *Ezrin*, which have significant roles in normal cellular processes and are overexpressed in aggressive cancers, indicates that miR-204 may control key regulatory mechanisms, dysregulation of which can predispose normal developmental/differentiation events to undergo transformation. Therefore, strategies aimed at using miR-204 as therapeutic regimens will have the advantage of reversing inappropriately activated steps in cancer cells to a more normal state. The identification of miR-204 as a potent tumor suppressor along with demonstration of its therapeutic potential and negligible hepatotoxicity establish a strong rationale for developing miR-204 as a viable therapeutic regimen to treat cancers.

## Materials and Methods

### Normal and Tumor Tissue Samples and Cell Lines

Cell lines representing pediatric renal tumors (HEK-293; embryonal kidney cell line), ovarian (SKOV3), and breast (MDA-MB-231) cancers used for experiments were obtained from ATCC and cultured according to the protocols provided. Seventy-six pediatric renal tumors and normal matched kidney were acquired from the Children’s Oncology Group (Arcadia, CA). Eleven advanced stage ovarian tumors and five normal ovarian tissues were obtained from UT MD Anderson Cancer Center (Houston, TX). Fifteen breast cancer tissues and normal matched tissues were obtained from UT Health Science Center (San Antonio, TX).

### RNA and Protein Analyses

Total RNA was extracted from tumors and normal tissues, as well as all cell lines, and was subjected to qRT-PCR analysis, as described previously [43]. Western blot analysis was performed as described previously [43]. Antibodies to β-actin (AC-74-A5316) and α-tubulin (T6199) were purchased from Sigma Aldrich. Antibody to BDNF was purchased from Santa Cruz, other antibodies including caspase-8 (IC12-9746), caspase-3 (8G10-9665), caspase-9 (9502), PARP (9542S), AKT (9272), phospho-AKT (9271), S6 ribosomal protein (2217), and phospho-S6 ribosomal protein (5G10-2211S) were purchased from Cell Signaling.

### Genomic PCR Assay

Genomic DNA from pediatric renal tumors (*n* = 38), and advanced stage (III and IV) ovarian cancers and matched normal renal (*n* = 38) and ovarian (*n* = 4) tissues were isolated with the DNeasy Genomic DNA Extraction Kit (Qiagen). For genomic analysis of miR-204, the 2^−ΔΔCt^ method was adapted using SYBR Green-based quantitative PCR (qRT-PCR) as described [3]. Briefly, 25 ng of genomic DNA was used as template to amplify the miR-204 locus. A representative control tissue sample was used in every assay as a calibrator to which every sample was compared, to obtain a relative quantitation (RQ) value. We used genomic DNA from a normal male sample (1x) and a normal female sample (2x) (Promega) to compare the levels of phosphoglycerate kinase 1 (PGK1) and five X-chromosome specific miRNAs – miR-424, miR-503, miR-766, miR-222 and miR-221– to calibrate the extent of loss in the various miRNA loci. RQ values for these regions in male genomic DNA were assessed using the non-X-chromosome TATA binding protein (TBP) gene as the reference. Based on its ability to separate two distinct data populations, an RQ value of 0.5 and below was considered as loss of at least one copy of a genomic locus.

### High-resolution miRNA Comparative Genomic Hybridization Assay (CGH)

MiRNA CGH analysis was performed on the genomic DNA isolated from pediatric renal tumor samples using an Agilent custom-designed microarray as per the manufacturer’s protocol. Custom arrays were designed based on the Agilent 2×105K Human Whole Genome Genomic Microarray using Agilent’s eArray program (https://earray.chem.agilent.com/earray), with additional probes that cover all miRNA regions (200 bp before, within and after of each miRNA from miRBase v13, with triplicate probes to enhance reliability). Array CGH data analysis was performed with Nexus Copy Number (BioDiscovery) for DNA alteration quantification. Upon determination of samples with or without copy number alteration at specific miRNA sites, all array CGH data was loaded into MATLAB for a composite graphical display. Array CGH showing position of the probes and normalized copy numbers have been submitted to GEO/NCBI data base (GSE28397).

### Meta-analysis

Meta-analysis was performed on a public domain high density CGH dataset on 354 ovarian cancers (obtained from The Cancer Genome Atlas Project (TCGA, http://cancergenome.nih.gov)) and 35 breast cancers (GSE15130). Array CGH data for all tumors imported into Nexus Copy Number. The threshold for copy number loss was set to log_2_ −0.5 (more stringent than the default setting of log_2_ −0.2). Meta-analysis for gene expression analysis was performed on a public domain gene expression dataset (GSE22820) from the GEO/NCBI. Differential expression of TRPM3 (the locus containing miR-204) in breast cancer samples was determined by first performing RMA/quantile normalization and then comparing to normal adjacent tissues within the same data set.

### Gene Expression Analysis

Human gene expression microarray data were generated using the Agilent Human Whole Genome 4×44K array (Agilent Technologies). Total RNA isolated from HEK-293 cells transfected with miR-204 was co-hybridized with RNA from HEK-293 cell transfected with scrambled oligo (control) with dye-swap replicates, following the manufacturer’s protocol. Relative gene expression ratios were extracted with Agilent’s Feature Extraction software (version 9.5.3.1, Agilent Technologies). Quantile normalization was performed with the MATLAB Bioinformatics Toolbox (R2009a, Mathworks). To determine differential expression of genes, we applied the Student’s *t*-test to the normalized expression data. Signature gene sets were selected based on an FDR-adjusted *P*<0.05 (Benjamini-Hochberg) and a fold change >2. Differential gene expression dataset has been deposited in GEO/NCBI data base (GSE 28400).

### Plasmid

For the pMIR-BDNF 3′ UTR construct, the 3′ UTR segment of the BDNF gene was amplified and sub-cloned downstream of the luciferase gene in the pMIR-REPORT vector (Ambion) at the *HindIII* and *SpeI* sites. For the pSilencer-miR-204 construct, ∼500 bp pri-miR-204 genomic sequence was amplified and cloned into the *BamHI* and *HindIII* sites of the pSilencer 4.1 Puro vector (Ambion). Constitutively active AKT T308D/S473D mutant (Plasmid 14751) was purchased from Addgene Inc.

### Cell Growth and Soft Agar Assay

Cell growth and soft agar colony formation assay using pSilencer-miR-204 and pSilencer-scramble (control) stable cell lines were performed as described previously [43].

### Tumorigenicity Assays in SCID mice

Two million HEK-293 cells stably overexpressing either pSilencer-miR-204 or pSilencer-scramble were injected into the renal capsules of 9 *RAG2* ^−/−^, γc^−/−^ SCID male mice (Taconic) and 9 control mice. Tumor volume was assessed 24 d after transplantation, using the formula π/6×(L×D×W), where L is tumor length, D is depth and W is width. Six micrometer paraffin embedded tumor sections were stained with hematoxylin and eosin. All experimental procedures involving animals were performed according to institutional ethical guidelines.

### Transwell Cell Migration and Basement Membrane Matrix Invasion Assay

Transwell cell migration assays were performed as previously described [43]. Invasion assays were performed with MDA-MB-231 cells transfected with 75 nM miR-204 mimic or negative control mimic, then further transfected for 48 h with 75 nM miR-204-specific inhibitor or 25 ng of pBluescript KS^+^ control, as described below. Forty-eight hours post-transfection, cells were seeded onto the inserts of 24-well transwell plates precoated with Matrigel (1 mg/mL) (BD Biosciences). Serum-containing media and fibronectin (5 µg/mL) were added to the lower chamber as chemoattractants. After 24 h incubation, cells on transwell inserts were washed, fixed, and non-invading cells and EC matrix were gently removed with a cotton swab. Invasive cells located on the lower side of the chamber were stained with crystal violet (0.1%), air dried and photographed as described [43].

### Therapeutic Experiments

We injected 100,000 MDA-MB-231-GFP-luc cells (kindly provided by Dr. Luzhe Sun, UTHSCSA, San Antonio, TX) into the tail vein. Starting from 7 d after tumor cell injection we injected miR-204 (*n* = 6) or miR-204 mutant (*n* = 6) oligos complexed with RNALancerII *in vivo* delivery formulation (Bioo Scientific) every 5 d for 30 d at a rate of 1 mg of oligo per kg of body weight. All animals were sacrificed after the sixth injection. Lungs were fixed and analyzed for metastatic foci. Lung images were captured using fluorescence microscope (for GFP^+ve^ and luc^+ve^ foci). We also harvested livers to assess metastasis and hepatoxicity in miR-204 injected mice.

### Apoptosis Assays

Annexin V/PI staining on HEK-293 or HeLa cells transfected with 75 nM miR-204 mimic or negative control mimic was performed in triplicate wells using the FITC-Annexin V Apoptosis Detection Kit (BD Pharmingen) as described previously [43].

### Immunofluorescence

Cells were fixed with 4% paraformaldehyde and permeabilized with 0.2% Triton X-100 for 15 min at room temperature (RT), then washed with phosphate-buffered saline (PBS) and blocked with 10% goat serum in PBS for 45 min at RT. Cells were incubated with Rac1 mouse monoclonal antibody (ab33186, Abcam) diluted in PBS for 2 h at RT. Cells were washed three times before incubating for 1 h at RT with 1∶200 Alexa Fluor 647 and Alexa Fluor 488 phalloidin-conjugated secondary antibodies (A12379, Invitrogen, Molecular Probes). After three washes, cells were mounted on glass slides in Aqua-Poly/Mount medium containing DAPI (Polysciences). Photomicrographs were taken at 200x magnification on a Nikon Eclipse TE2000-U microscope.

### Statistical Analysis

All values and error bars in graphs are means ± SEM; respective *n* values are indicated in figure legends; *P*-values are determined by two-tailed Student’s *t*-tests.

## Supporting Information

Figure S1 Copy number loss of chromosomal regions containing miRNAs in tumors. A, list of miRNAs (obtained from high resolution miRNA CGH (pediatric renal tumors and ovarian cancers) and meta-analysis of public domain CGH database (ovarian cancers and breast cancers) showing copy number loss in all three tumors. B, graphs obtained from meta-analysis of high-resolution CGH of breast cancers (*n* = 35; GSE 15130) represent a subset of tumors with or without deletion.
(TIF)

Figure S2 Systemic delivery of miR-204 suppresses tumor metastasis. A, live bioluminescence images of mice injected with miR-204 or miR-204 mutant (control oligo injected) oligonucleotides using IVIS. Images were taken after subcutaneously injecting 150 mg/kg D-luciferin substrate in PBS to anesthetized mice. B, representative lung sections showing metastatic foci only in neg. control group.
(TIF)

Figure S3 MiR-204 targets genes associated with tumorigenesis. A, list of downregulated genes (≥2-fold threshold) obtained from microarray analyses on scramble or miR-204 overexpressing cells. B, biological function analyses of downregulated genes (≥2-fold threshold) obtained from microarray analyses on cells overexpressing miR-204 using Ingenuity pathway analyses software. MiR-204 target genes associated with biological functions and canonical pathways in the Ingenuity Pathways Knowledge Base were considered for the analysis. Fischer’s exact test was used to calculate a p-value determining the probability that each biological function and canonical pathway assigned to the candidate miRNA target genes is due to chance alone. Biological functions and canonical pathways with a *P*<0.05 were considered significant. C, network analyses of downregulated genes (≥2-fold threshold) obtained from microarray analyses on HEK-293 cells overexpressing miR-204 using Ingenuity pathway analyses software. Note that several of these network genes including BDNF and Ezrin are known to be involved in tumorigenesis. Microarray dataset is deposited in the GEO data base (GSE28397).
(TIF)

Figure S4 MiR-204 regulates expression of Ezrin in cancers. Increased Ezrin expression correlates strongly with lower miR-204 expression in multiple cancers. Graphical representation of qRT-PCR analysis showing the inverse correlation between miR-204 and *Ezrin* in pediatric renal tumors (*n* = 38; A), advanced stage ovarian cancers (*n* = 11; B) and breast cancers (*n* = 10; C), compared to normal matched control kidney (*n* = 38), normal ovarian tissues (*n* = 5) and normal matched breast tissues (*n* = 10). D–F, *Ezrin* is a bona fide target of miR-204. D, schematic of the putative miR-204 binding sequence in the *Ezrin* 3′ UTR (top). HEK-293 cells were co-transfected with Renilla luciferase expression construct pRL-TK and firefly luciferase constructs containing pMIR-*Ezrin* 3′ UTR in the absence and presence of miR-204 mimic (bottom). Firefly luciferase activity of each sample was normalized to Renilla luciferase activity. Mean±SEM of three independent experiments (performed in duplicate for each experiment). (**) *P*<0.01; (***) *P*<0.001. E, qRT-PCR analysis of cells transfected with scramble or miR-204 mimic using *Ezrin*-specific primers. F, western blot analysis of HEK-293 cells transfected with miR-204 mimic using anti-Ezrin antibody (1∶2500). β-actin was used as a loading control. Gel photographs are representative of three independent experiments. Values below the gel were quantified using the Total Labs TL100 1D gel analysis software. Ezrin protein level for the control was set to one.
(TIF)

Figure S5 BDNF rescues miR-204 associated phenotypes. A, western blot analysis of ovarian cancer (SKOV3) and breast cancer (MDA-MB-231) cells transfected with miR-204 mimic or miR-204 inhibitors using anti-BDNF antibody (1∶1000). β-actin was used as a loading control. Gel photographs are representative of three independent experiments. B and C, BDNF rescues miR-204 mediated inhibition of cell migration and invasion. Migration (B) and invasion (C) ability of MDA-MB-231 cells transfected with either 10 nM negative control miRNA (control), miR-204 mimic (miR-204) or miR-204 mimic and *BDNF* (miR-204+BDNF). Photomicrographs represent three independent experiments (in triplicate wells). Bar graph showing average number of migrated/invaded cells counted microscopically (200x) in five different fields per filter. (***) *P*<0.001; (**) *P*<0.01.
(TIF)

Figure S6 MiR-204 targets AKT/mTOR pathway. A and B, immunohistochemical analysis on miR-204 overexpressing and control transfectant tumor xenograft sections using anti-phospho-Ser^473^-AKT (1∶50; A, top), anti-total-AKT (1∶50; A, bottom), anti-phospho-Ser^235/236^-S6 (1∶100; B, top) and anti-total-S6 (1∶100; B, bottom) antibodies.
(TIF)

Figure S7 Overexpression of miR-204 abolishes EGF-induced membrane ruffling and Rac1 translocation. SKOV3 cells transfected with miR-204 were grown in serum free condition and treated with or without EGF (100 ng/ml) for 10 min and stained with Rac1 antibody (red) and FITC-phalloidin (green). Arrows indicate membrane-ruffling regions.
(TIF)

Figure S8 MiR-204 does not affect *TRPM3* levels. QRT-PCR analysis of HEK-293 cells transfected with scramble, miR-204 mimic or miR-204 inhibitor using *TRPM3*-specific primers.
(TIF)
