# Supplementary figures and images for: Genomic Loss of Tumor Suppressor miRNA-204 Promotes Cancer Cell Migration and Invasion by Activating AKT/mTOR/Rac1 Signaling and Actin Reorganization
Source: PLoS One. 2012 Dec 21;7(12):e52397. doi: 10.1371/journal.pone.0052397 (PMC3528651; doi:10.1371/journal.pone.0052397)

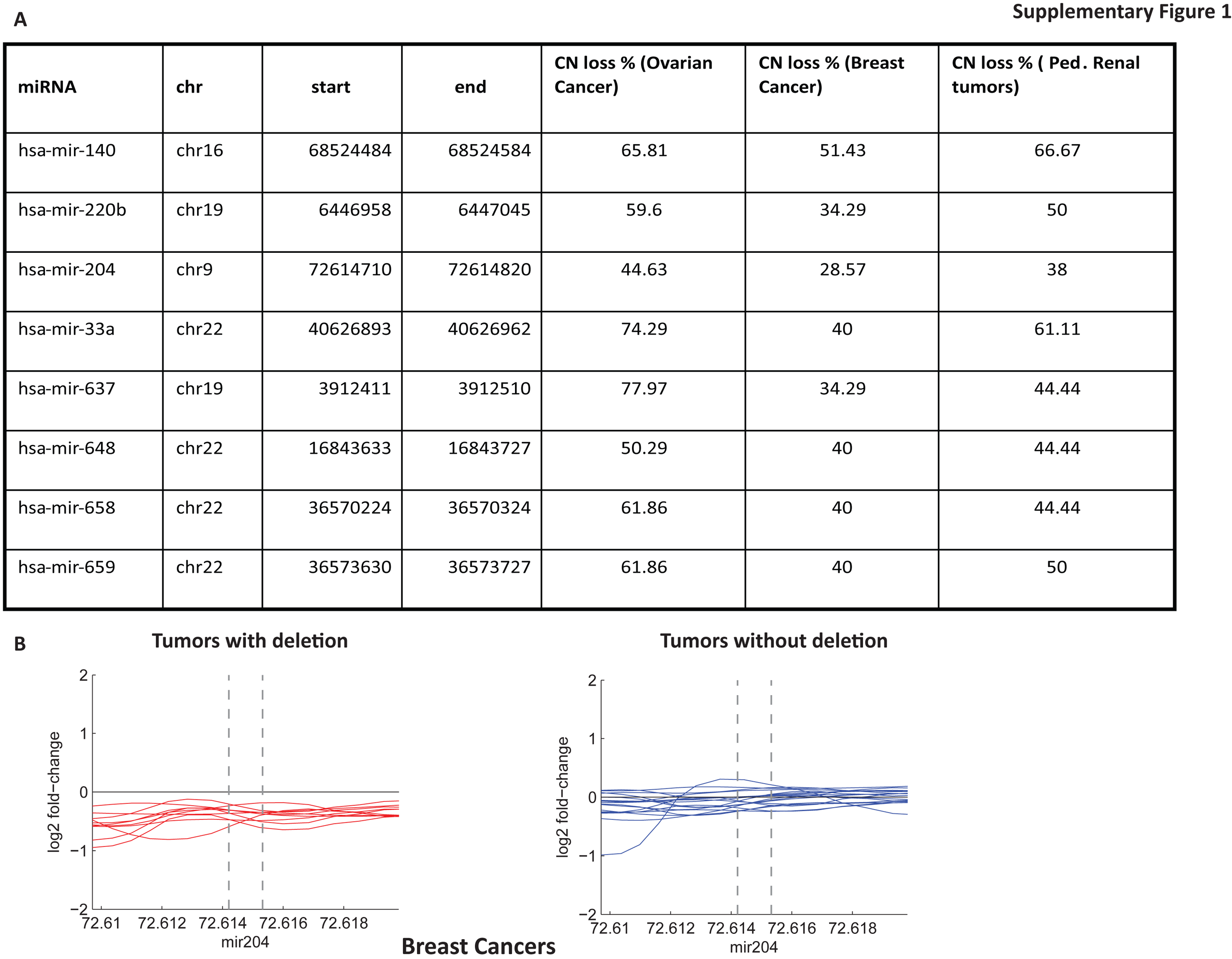

Supplement: Figure S1 — Copy number loss of chromosomal regions containing miRNAs in tumors. A, list of miRNAs (obtained from high resolution miRNA CGH (pediatric renal tumors and ovarian cancers) and meta-analysis of public domain CGH database (ovarian cancers and breast cancers) showing copy number loss in all three tumors. B, graphs obtained from meta-analysis of high-resolution CGH of breast cancers (n = 35; GSE 15130) represent a subset of tumors with or without deletion. (TIF) [file pone.0052397.s001.tif]

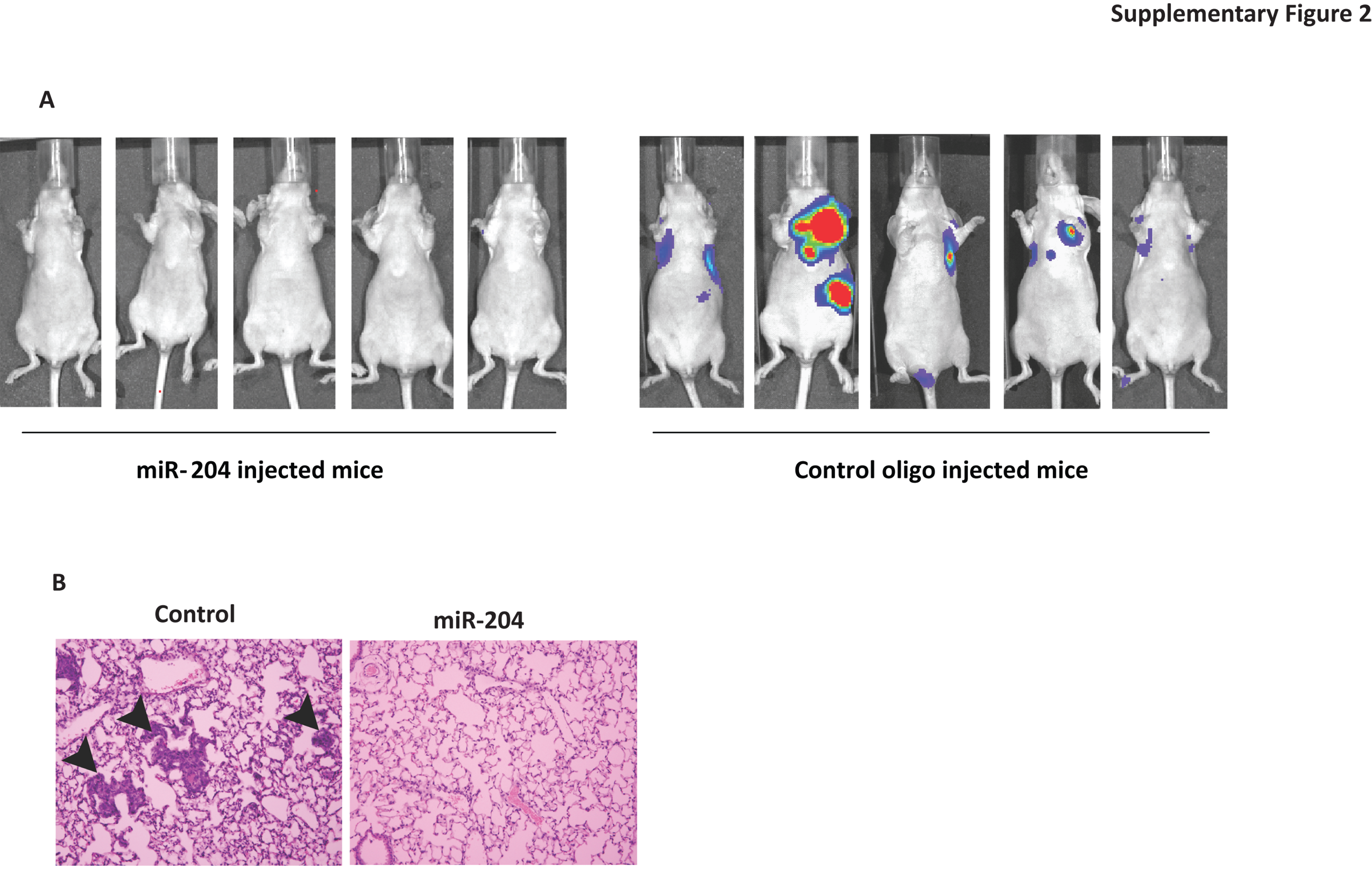

Supplement: Figure S2 — Systemic delivery of miR-204 suppresses tumor metastasis. A, live bioluminescence images of mice injected with miR-204 or miR-204 mutant (control oligo injected) oligonucleotides using IVIS. Images were taken after subcutaneously injecting 150 mg/kg D-luciferin substrate in PBS to anesthetized mice. B, representative lung sections showing metastatic foci only in neg. control group. (TIF) [file pone.0052397.s002.tif]

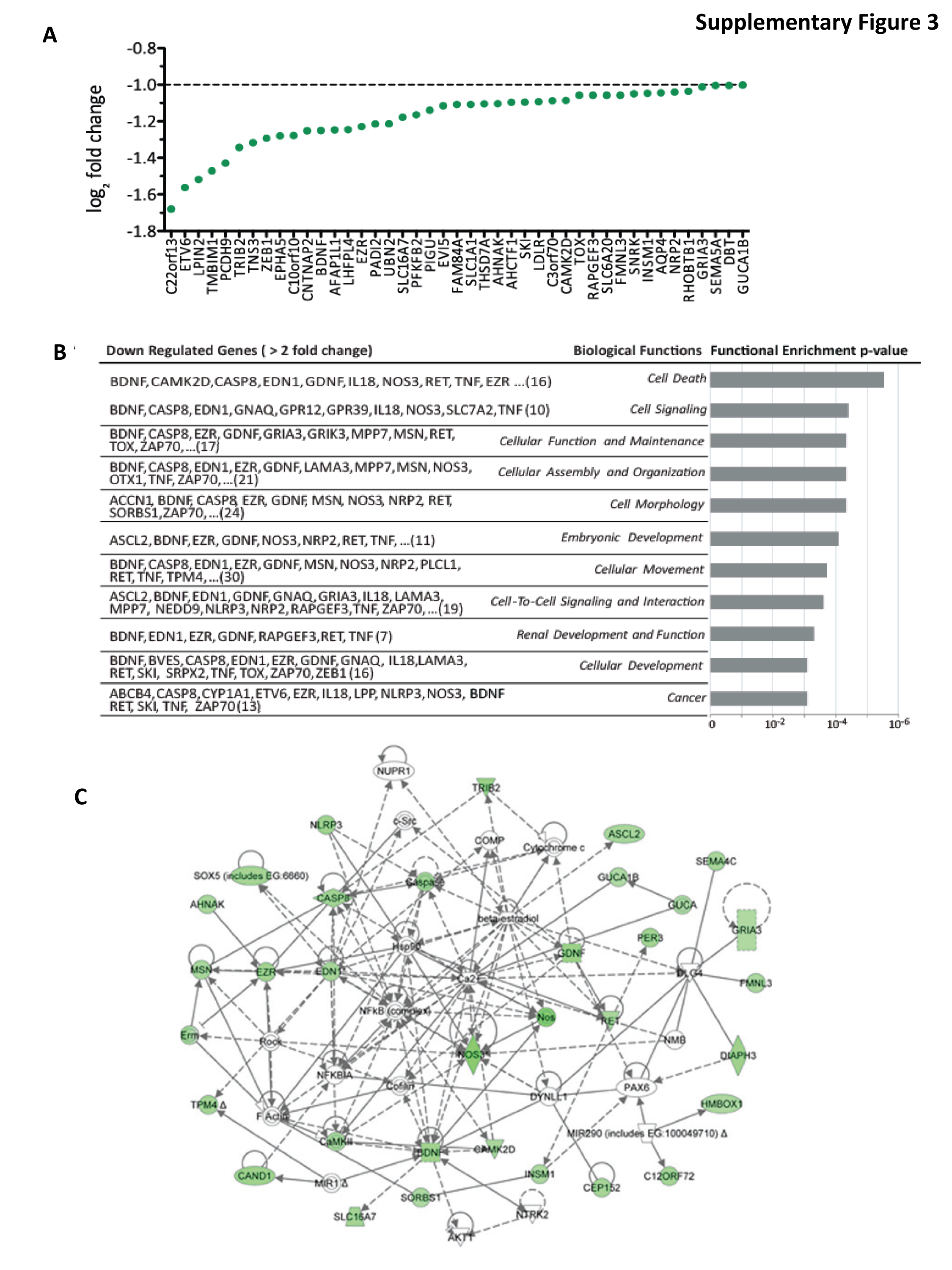

Supplement: Figure S3 — MiR-204 targets genes associated with tumorigenesis. A, list of downregulated genes (≥2-fold threshold) obtained from microarray analyses on scramble or miR-204 overexpressing cells. B, biological function analyses of downregulated genes (≥2-fold threshold) obtained from microarray analyses on cells overexpressing miR-204 using Ingenuity pathway analyses software. MiR-204 target genes associated with biological functions and canonical pathways in the Ingenuity Pathways Knowledge Base were considered for the analysis. Fischer’s exact test was used to calculate a p-value determining the probability that each biological function and canonical pathway assigned to the candidate miRNA target genes is due to chance alone. Biological functions and canonical pathways with a P<0.05 were considered significant. C, network analyses of downregulated genes (≥2-fold threshold) obtained from microarray analyses on HEK-293 cells overexpressing miR-204 using Ingenuity pathway analyses software. Note that several of these network genes including BDNF and Ezrin are known to be involved in tumorigenesis. Microarray dataset is deposited in the GEO data base (GSE28397). (TIF) [file pone.0052397.s003.tif]

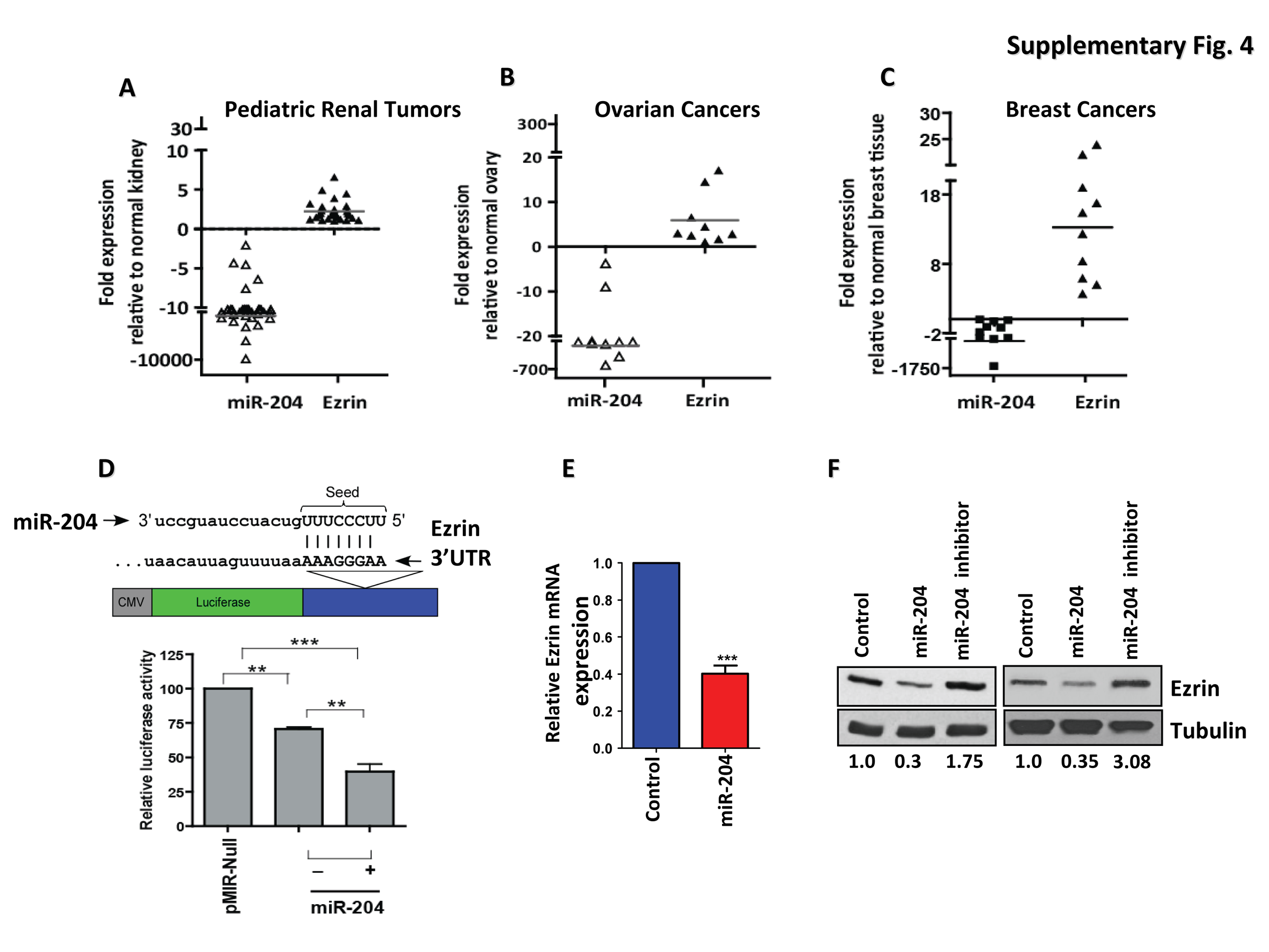

Supplement: Figure S4 — MiR-204 regulates expression of Ezrin in cancers. Increased Ezrin expression correlates strongly with lower miR-204 expression in multiple cancers. Graphical representation of qRT-PCR analysis showing the inverse correlation between miR-204 and Ezrin in pediatric renal tumors (n = 38; A), advanced stage ovarian cancers (n = 11; B) and breast cancers (n = 10; C), compared to normal matched control kidney (n = 38), normal ovarian tissues (n = 5) and normal matched breast tissues (n = 10). D–F, Ezrin is a bona fide target of miR-204. D, schematic of the putative miR-204 binding sequence in the Ezrin 3′ UTR (top). HEK-293 cells were co-transfected with Renilla luciferase expression construct pRL-TK and firefly luciferase constructs containing pMIR-Ezrin 3′ UTR in the absence and presence of miR-204 mimic (bottom). Firefly luciferase activity of each sample was normalized to Renilla luciferase activity. Mean±SEM of three independent experiments (performed in duplicate for each experiment). (**) P<0.01; (***) P<0.001. E, qRT-PCR analysis of cells transfected with scramble or miR-204 mimic using Ezrin-specific primers. F, western blot analysis of HEK-293 cells transfected with miR-204 mimic using anti-Ezrin antibody (1∶2500). β-actin was used as a loading control. Gel photographs are representative of three independent experiments. Values below the gel were quantified using the Total Labs TL100 1D gel analysis software. Ezrin protein level for the control was set to one. (TIF) [file pone.0052397.s004.tif]

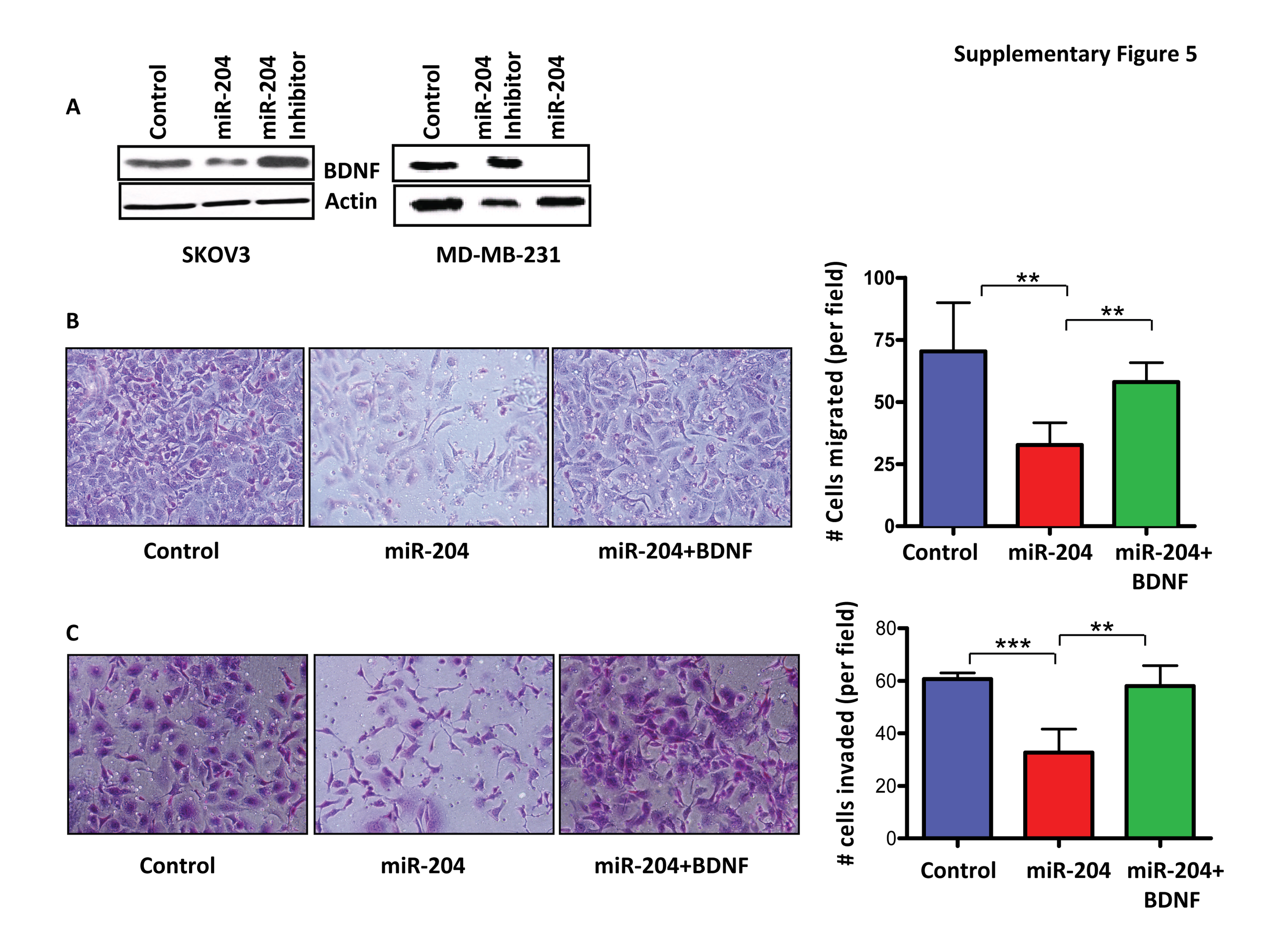

Supplement: Figure S5 — BDNF rescues miR-204 associated phenotypes. A, western blot analysis of ovarian cancer (SKOV3) and breast cancer (MDA-MB-231) cells transfected with miR-204 mimic or miR-204 inhibitors using anti-BDNF antibody (1∶1000). β-actin was used as a loading control. Gel photographs are representative of three independent experiments. B and C, BDNF rescues miR-204 mediated inhibition of cell migration and invasion. Migration (B) and invasion (C) ability of MDA-MB-231 cells transfected with either 10 nM negative control miRNA (control), miR-204 mimic (miR-204) or miR-204 mimic and BDNF (miR-204+BDNF). Photomicrographs represent three independent experiments (in triplicate wells). Bar graph showing average number of migrated/invaded cells counted microscopically (200x) in five different fields per filter. (***) P<0.001; (**) P<0.01. (TIF) [file pone.0052397.s005.tif]

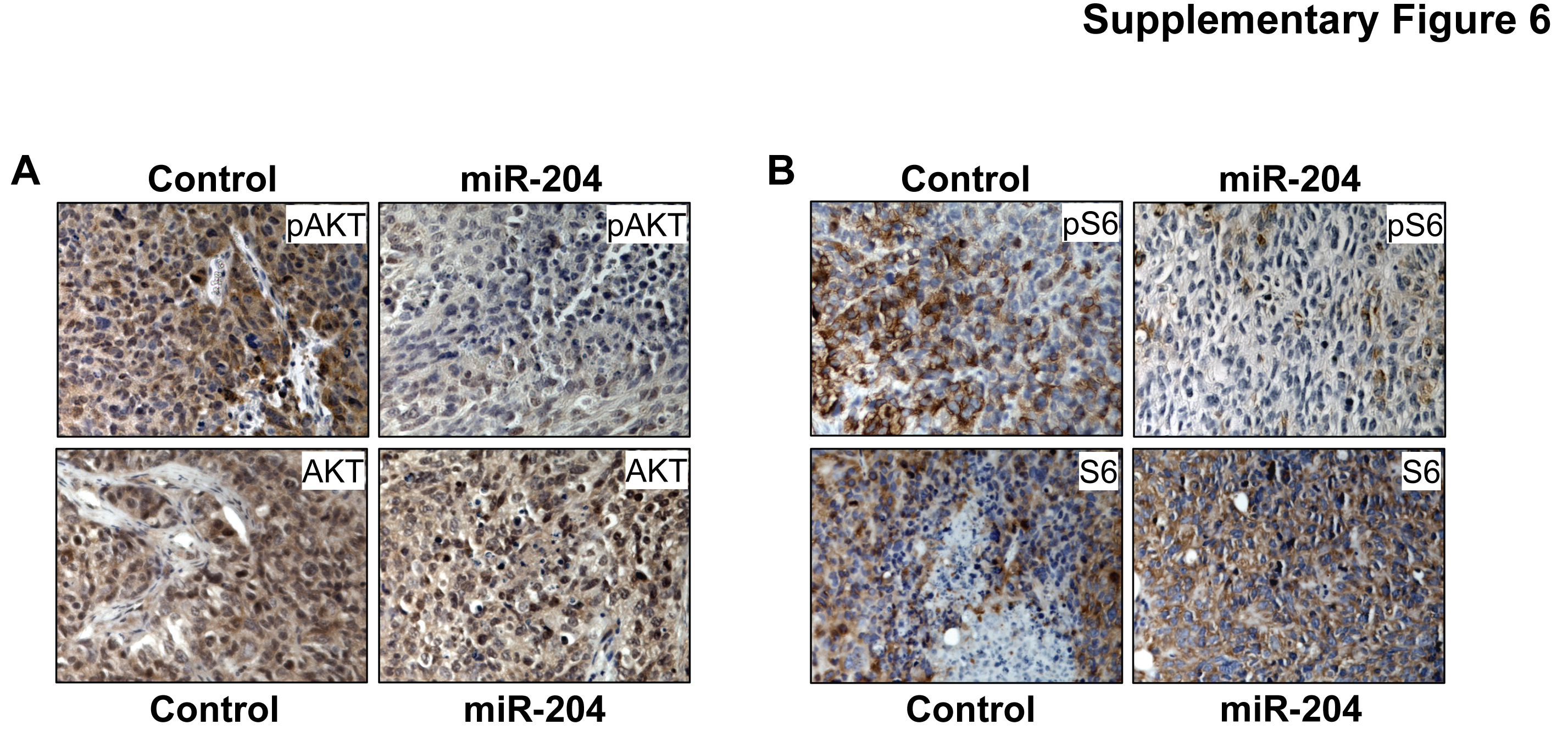

Supplement: Figure S6 — MiR-204 targets AKT/mTOR pathway. A and B, immunohistochemical analysis on miR-204 overexpressing and control transfectant tumor xenograft sections using anti-phospho-Ser473-AKT (1∶50; A, top), anti-total-AKT (1∶50; A, bottom), anti-phospho-Ser235/236-S6 (1∶100; B, top) and anti-total-S6 (1∶100; B, bottom) antibodies. (TIF) [file pone.0052397.s006.tif]

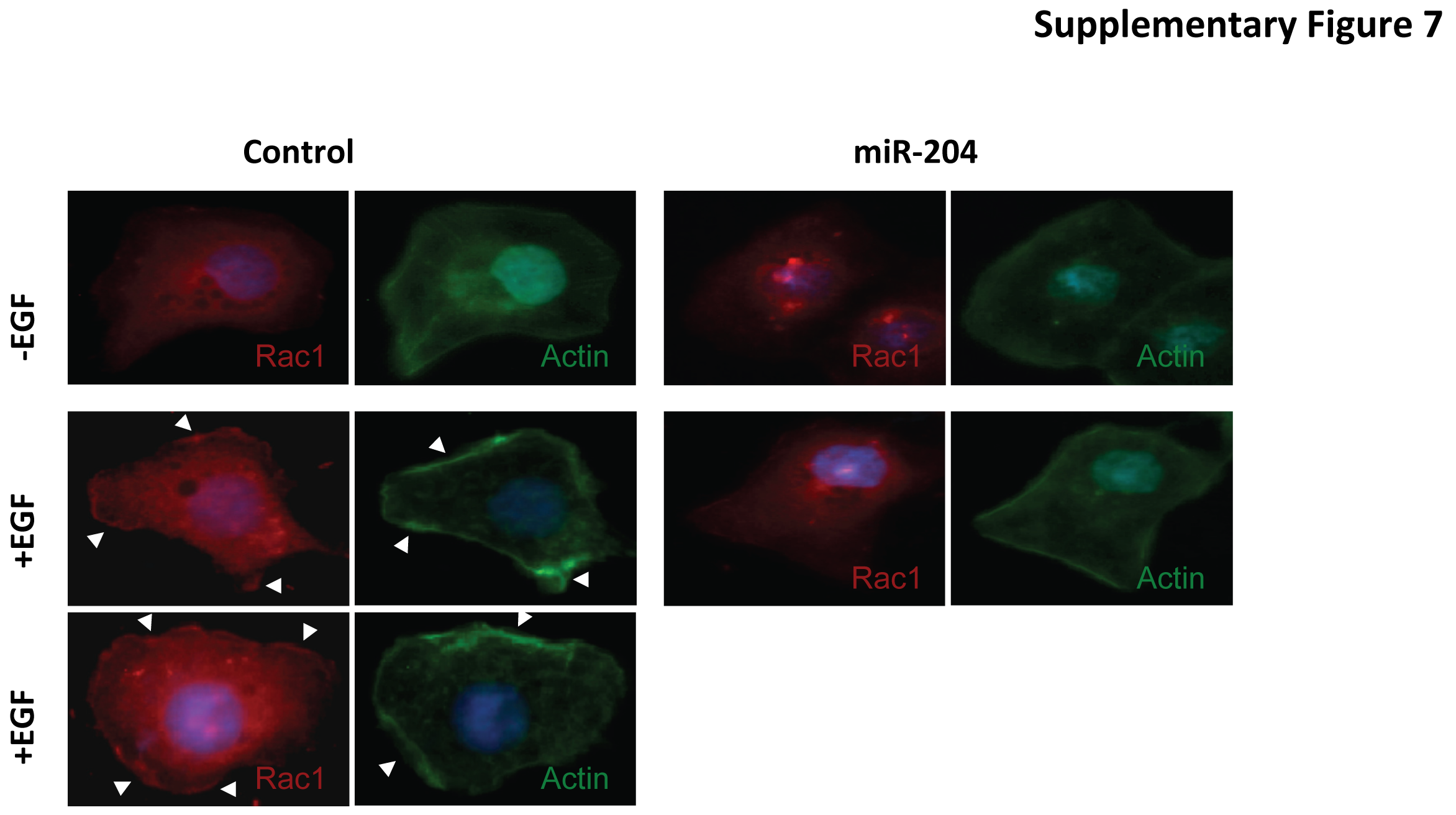

Supplement: Figure S7 — Overexpression of miR-204 abolishes EGF-induced membrane ruffling and Rac1 translocation. SKOV3 cells transfected with miR-204 were grown in serum free condition and treated with or without EGF (100 ng/ml) for 10 min and stained with Rac1 antibody (red) and FITC-phalloidin (green). Arrows indicate membrane-ruffling regions. (TIF) [file pone.0052397.s007.tif]

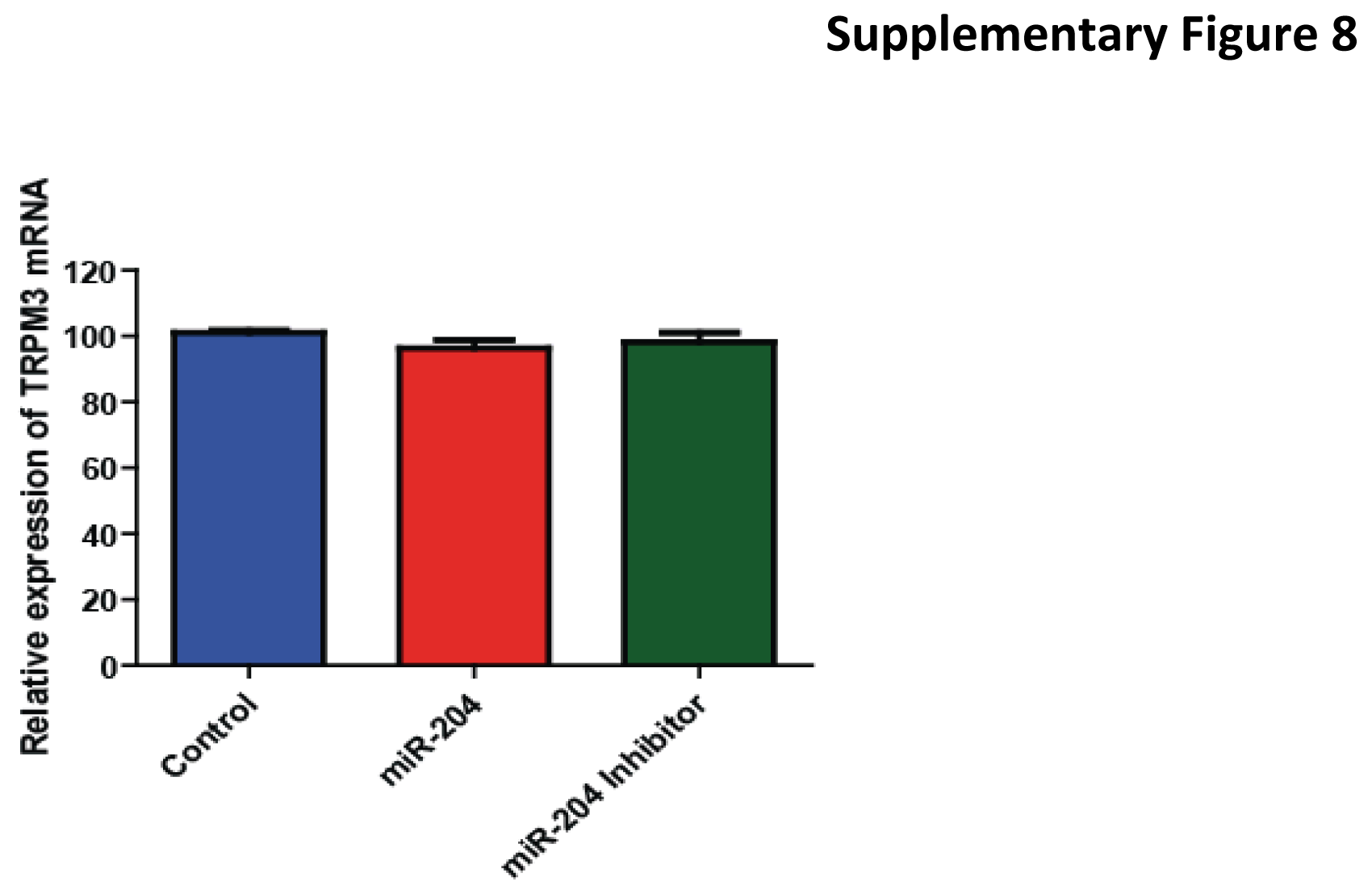

Supplement: Figure S8 — MiR-204 does not affect TRPM3 levels. QRT-PCR analysis of HEK-293 cells transfected with scramble, miR-204 mimic or miR-204 inhibitor using TRPM3-specific primers. (TIF) [file pone.0052397.s008.tif]
